# Prospective Associations of Maternal Dietary Patterns and Postpartum Mental Health in a Multi-Ethnic Asian Cohort: The Growing up in Singapore towards Healthy Outcomes (GUSTO) Study

**DOI:** 10.3390/nu10030299

**Published:** 2018-03-02

**Authors:** Cherlyen Teo, Ai-Ru Chia, Marjorelee T. Colega, Ling-Wei Chen, Doris Fok, Wei Wei Pang, Keith M. Godfrey, Kok Hian Tan, Fabian Yap, Lynette Pei-Chi Shek, Yap-Seng Chong, Michael Meaney, Helen Chen, Mary Foong-Fong Chong

**Affiliations:** 1 Singapore Institute for Clinical Sciences, Agency for Science, Technology and Research, Singapore 117609, Singapore; cherlyen_teo@sics.a-star.edu.sg (C.T.); marjorelee_colega@sics.a-star.edu.sg (M.T.C.); paeshekl@nus.edu.sg (L.P.-C.S.); yap_seng_chong@sics.a-star.edu.sg (Y.-S.C.); michael_meaney@sics.a-star.edu.sg (M.M.); 2 Department of Obstetrics & Gynaecology, Yong Loo Lin School of Medicine, National University of Singapore and National University Health System, Singapore 119228, Singapore; chiaairu@u.nus.edu (A.-R.C.); obglnld@nus.edu.sg (D.F.); obgpww@nus.edu.sg (W.W.P.); 3 Department of Paediatrics, Yong Loo Lin School of Medicine, National University of Singapore, Singapore 119228, Singapore; ling-wei.chen@ucd.ie; 4 School of Public Health, Physiotherapy and Sports Science, University College Dublin, Dublin 4, Ireland; 5 Medical Research Council Lifecourse Epidemiology Unit and National Institute for Health Research Southampton Biomedical Research Centre, University of Southampton and University Hospital Southampton National Health Service Foundation Trust, Southampton SO16 6YD, UK; kmg@mrc.soton.ac.uk; 6 Duke-National University of Singapore Graduate Medical School, Singapore 169857, Singapore; tan.kok.hian@singhealth.com.sg (K.H.T.); fabian.yap.k.p@singhealth.com.sg (F.Y.); helen.chen.y@kkh.com.sg (H.C.); 7 Department of Maternal Fetal Medicine, KK Women’s and Children’s Hospital, Singapore 229899, Singapore; 8 Department of Paediatric Endocrinology, KK Women’s and Children’s Hospital, Singapore 229899, Singapore; 9 Lee Kong Chian School of Medicine, Nanyang Technological University, Singapore 308232, Singapore; 10 Douglas Mental Health University Institute, McGill University, Montreal, QC H4H 1R3, Canada; 11 Department of Psychological Medicine, KK Women’s & Children’s Hospital, Singapore 229899, Singapore; 12 Saw Swee Hock School of Public Health, National University of Singapore and National University Health System, Singapore 117549, Singapore

**Keywords:** postpartum depression, postpartum anxiety, maternal diet, confinement diet, dietary patterns

## Abstract

Diet in the first month postpartum, otherwise known as “the confinement diet” in Asia, has unique characteristics that are influenced by traditions, cultures, and beliefs. We aimed to characterize dietary patterns during confinement period in a multi-ethnic Asian cohort and examined their associations with postpartum depression (PPD) and anxiety (PPA). Dietary intakes of 490 women were ascertained in the first month postpartum using 3-day food diaries and dietary patterns were derived by factor analysis. Participants completed the Edinburgh Postnatal Depression Scale (EPDS) and State-Trait Anxiety Inventory (STAI) at three months’ postpartum; higher scores are indicative of more depressive and anxiety symptoms, respectively. Four dietary patterns were identified: Traditional-Chinese-Confinement diet, Traditional-Indian-Confinement diet, Eat-Out diet and Soup-Vegetables-Fruits diet. The Traditional-Indian-Confinement diet was associated with less PPD symptoms [β (95% CI) −0.62 (−1.16, −0.09) EPDS score per SD increase in diet score] and a non-significant trend with reduced probable PPD (EPDS scores ≥ 13) [OR (95% CI) 0.56 (0.31, 1.01)]. The Soup-Vegetables-Fruits diet was associated with less PPA symptoms [β (95% CI) −1.49 (−2.56, −0.42) STAI-state score]. No associations were observed for other dietary patterns. Independent of ethnicity, adherence to the Traditional-Indian-Confinement diet that is characterized by intake of herbs and legumes, and Soup-Vegetables-Fruits diet high in fruits, vegetables and fish during the postpartum period were associated with less PPD and PPA symptoms, respectively.

## 1. Introduction

Postpartum depression (PPD) and postpartum anxiety (PPA) affect 13–19% [1,2] of women worldwide, respectively. The impact of these disorders can range from immediate effects of self-harm, inadequate caregiving practices, reduced breastfeeding, to long-term consequences, such as maternal chronic depression and child temperament, cognitive, and behavioral issues [2,3,4,5]. These disorders often go undiagnosed and untreated due to the lack of awareness of symptoms and the associated stigma [6], thus preventive measures need to be identified to mitigate their risks. 

Nutrition has been shown to play roles in the prevention of mental health disorders through its involvement in the synthesis and regulation of neurotransmitters, neuronal membrane fluidity, synaptic plasticity and brain functions implicated in the etiology of depression and anxiety [7]. In the post-parturition period, women are more susceptible to nutrient deficiencies due to delayed postpartum repletion and lactation [8]. For example, low levels of nutrients, such as zinc, magnesium, and n-3 fatty acids have been linked to PPD [7,9,10,11,12] and are associated with anxiety in general population and animal studies [13,14,15]. 

The synergistic and antagonistic interactions among nutrients and the recognition that foods are often consumed in combination has led to an increasing use of dietary patterns to examine the associations between diet and health outcomes [16,17,18,19,20,21,22,23]. Dietary patterns are also able to capture food preferences of individuals that are influenced by genetic, cultural, and lifestyle factors [24]. Studies have demonstrated associations between dietary patterns during the pre-pregnancy or pregnancy periods and maternal mental health disorders. A healthy dietary pattern that is characterized by higher intakes of vegetables, fruits, and fish during pre-pregnancy [22], and pregnancy [17] have been reported as protective against perinatal anxiety symptoms. While two cohort studies did not find any association between healthy diet during pregnancy and risk of PPD [20,21], the Rhea mother-child study in Greece found that adherence to “health-conscious” dietary pattern during pregnancy was associated with a lower risk of PPD [19]. 

The literature examining potential associations between postnatal dietary patterns and postpartum mental health status remains limited. To our knowledge, only one study has observed poor diet quality at 6 to 12 months after delivery in relation to more depressive symptoms at one year postpartum in a group of 146 low-income women [25]. 

In Asia, the period 21 to 40 days after parturition is believed to be a period of convalescence and also known as “the confinement period”. During this period, mothers follow specific dietary and behavioral restrictions and prescriptions, which are aimed at promoting restoration of maternal health and to protect mothers from future illnesses [26,27]. These prescriptions are shaped by cultural beliefs [26,27,28,29] that have common origins. For example, the confinement practices of the three major Asian ethnic groups (i.e., Chinese, Malays and Indians) are built on hot-cold theory of diseases that emphasizes the maintenance of equilibrium of “hot” and “cold” in the body as essential to staying healthy [26,30,31]. The common belief is that parturition leaves the body in a “cold” state, thus “hot” foods are prescribed, while “cold” and raw foods are avoided during the confinement period [27,28,29,30]. However, food that is considered “hot” or “cold” varies across the three cultures, giving rise to different prescriptions and proscriptions of diet. Other behavioral prescriptions include not leaving the house, not washing one’s hair or shower, having traditional massages and abdominal binding throughout the confinement period [30]. 

Postnatal dietary patterns followed during confinement period in Asia have not been well characterized. While these traditional confinement practices have been passed down generations, little is known about its possible influence on maternal mental health. As a multi-cultural society encompassing three major ethnic groups of Chinese, Malay, and Indian, Singapore provides a suitable opportunity for studying postnatal dietary patterns followed during confinement period. In this study, we first derived postnatal dietary patterns of a multi-ethnic cohort of mothers and subsequently examined their associations with maternal mental health status at three months postpartum.

## 2. Materials and Methods

### 2.1. Study Design and Participants

Data were obtained from the Growing Up in Singapore Towards healthy Outcomes (GUSTO) study. The GUSTO study was set up to investigate the potential role of early-life influences in development of metabolic diseases [32]. This study was approved by the National Health Care Group Domain Specific Review Board and the Sing Health Centralized Institutional Review Board. 

Women who were attending their first antenatal dating scan (<13 weeks) at National University Hospital (NUH) and KK Women’s and Children’s Hospital (KKH) were recruited into the study between June 2009 and September 2010. To be eligible for inclusion in the study, participants had to be between 18 and 50 years of age, Singaporeans or permanent residents of Singapore, and from Chinese, Malay, or Indian ethnic group with homogeneous parental ethnic background. Only participants who had intended to deliver in NUH or KKH, reside in Singapore for the next five years, and who were willing to donate cord blood, cord, and placenta at delivery were included. Women who were undergoing chemotherapy treatment, using psychotropic drugs or with serious health issues, such as type 1 diabetes mellitus were not included in the study. Written informed consent was collected from each participant. 

### 2.2. Postnatal Dietary Intake

Postnatal dietary intake was assessed using 3-day food diaries, which were provided after delivery and collected at three weeks post-delivery. Participants were asked to record detailed descriptions of food and drinks they had consumed for three days (two weekdays and one weekend) during the postpartum period. The 3-day food diary included pictures depicting standardized household measuring utensils and various portion sizes of different food items to assist participants in quantifying the amount of food they have consumed. 

From the dietary records, weight of each food item consumed was determined using a food composition database of locally available foods [33], or the US national food composition database [34]. Composite dishes were broken down into individual food items according to the recipes in the database. All of the food items were assigned to one of 84 food groups (Supplementary Table S1), based on their similarity in nutritional characteristics and comparable usage (e.g., cabbage, spinach, tomatoes were grouped into the food group “Vegetables-Cruciferous, Leafy, Yellow/Orange/Red”). The average amount (g) of each food group consumed per day was tabulated for each subject.

### 2.3. Maternal Mental Health Status

To assess maternal mental health status, Edinburgh Postnatal Depression Scale (EPDS) and State-Trait Anxiety Inventory (STAI) were self-administered by the participants at 26–28 weeks of gestation and at three months postpartum. The EPDS questionnaire was developed to screen for postnatal depression [35] and has been widely used as a screening tool for PPD and antenatal depression across multiple cultures [36]. This instrument consists of ten four-point Likert scale items (0 to 3) where the response categories in each item are assigned scores of 0, 1, 2, and 3, according to increasing intensity of the symptoms. Participants were asked to select the response that best reflects their feelings for the past week and a total score was calculated. The total score of this questionnaire ranges from 0 to 30, with a higher score indicating more depressive symptoms. Cut-off scores of 14/15 and 12/13 were used to identify probable antenatal depression and probable PPD, respectively [36,37]. In this study, participants with EPDS scores ≥ 15 during pregnancy [38] and ≥13 at three months postpartum [35] were identified as having probable antenatal depression and probable PPD, respectively. 

Symptoms of anxiety were measured with 40-item STAI, which comprises of two subscales, the STAI-state, and STAI-trait. STAI-state was used as a continuous measure of current state of anxiety, while STAI-trait assessed longstanding trait of anxiety. In this study, only the STAI-state subscale was considered, as we were interested to consider how the current postpartum state was related to postnatal dietary patterns. The STAI-state subscale has proven stable internal consistency with Cronbach α value of 0.91 [39]. Subscale score can range from 20 to 80, with a higher score indicating more anxiety symptoms [40].

### 2.4. Covariates

Information on maternal age, ethnicity, education level, marital status, self-reported obstetric, and medical history was collected through interviewer-administered questionnaires during recruitment. Data on cigarette smoking, exposure to cigarette smoke, alcohol consumption before and during pregnancy, exercise extent, and employment status during pregnancy were captured with questionnaires at 26–28 weeks of gestation. In addition, maternal weight and standing height were measured using SECA weighing scale (803, SECA Corp., Hamburg, Germany) and SECA stadiometer (model 213), respectively. Maternal pregnancy Body Mass Index (BMI) was calculated as mother’s pregnancy weight divided by the square of mother’s height (kg/m^2^). During the same clinic visit, oral glucose tolerance tests were conducted and participants with Gestational Diabetes Mellitus (GDM) were diagnosed based on WHO diagnostic criteria [41]. Birth data such as gestational age and mode of delivery were extracted from delivery case notes while information on postpartum practices and availability of social support was obtained through questionnaires at three weeks post-delivery. The type of infant feeding (exclusively breastfed/partially breastfed/formula fed) for the first three weeks and 4th week of life were ascertained at week three and month three postpartum visit, respectively. The mode of infant feeding during 1st month postpartum was determined by computing the mode of type of feed across the first four weeks from birth.

### 2.5. Participants Included in Analysis

**Figure 1:**
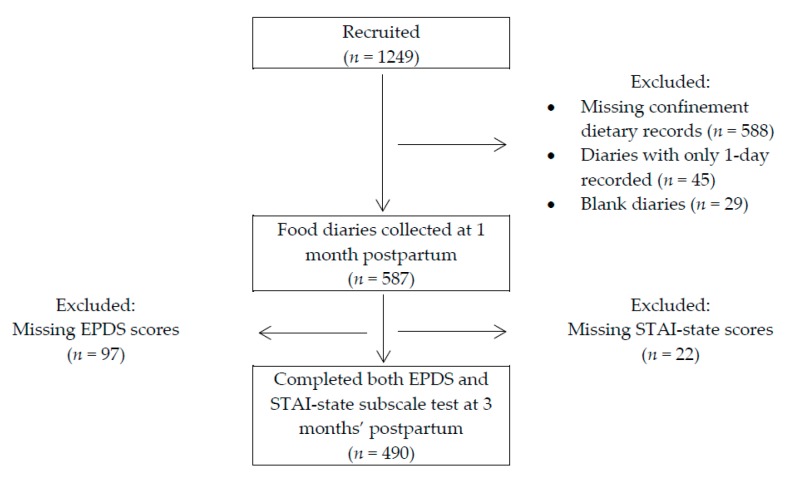
Flowchart showing selection of participants who had postpartum dietary records, Edinburgh Postnatal Depression Scale (EPDS) and State-Trait Anxiety Inventory (STAI)-state scores from Growing Up in Singapore Towards healthy Outcomes (GUSTO) study cohort.

Among 1249 women that were recruited in the study, 490 participants who provided postnatal 3-day food diary and completed EPDS and STAI at three months’ postpartum were included in this study (Figure 1). No significant differences were observed among participants included and excluded from the study in terms of parity. Participants included in the study tended to be older, of Chinese ethnicity, university graduates, have lower pregnancy BMI and were more likely to exclusively breastfeed their child during 1st month postpartum (Supplementary Table S2).

### 2.6. Derivation of Dietary Patterns

Dietary patterns were derived using exploratory factor analysis with principal component extraction and varimax rotation on 84 food groups (Table 1). Four factors were extracted based on break point of Scree plot and interpretability of factors. The dietary pattern score for each participant was calculated by summing the standardized intakes of food groups (g/day) weighted by their corresponding factor loadings. Factor loadings can be interpreted as the correlation coefficient of each food group and its corresponding factor (i.e., dietary pattern); hence, higher dietary pattern scores reflect greater adherence to the derived pattern. 

### 2.7. Statistical Analysis

Characteristics of participants were summarized according to tertiles of dietary pattern scores, while P-trends for association were assessed by modelling the median value of each tertile in linear regression analysis for continuous variables and Cochran-Mantel-Haenszel tests for categorical variables (Table 2 and Supplementary Table S3). 

To investigate the associations between postnatal dietary pattern scores and symptoms of PPD and PPA, EPDS and STAI-state scores obtained at three months’ postpartum were modelled continuously using linear regression, while likelihood of probable PPD (EPDS score ≥ 13) was evaluated using logistic regression. The models were adjusted for maternal age, ethnicity, parity, education level, history of miscarriage, probable depression of mothers at 26 weeks gestation (for EPDS scores and probable PPD models), STAI-state score at 26 weeks gestation (for STAI-state score model), and mode of infant feeding during 1st month postpartum.

Data were missing for the following variables: education level, *n* = 5; probable depression at 26 weeks gestation, *n* = 9; high level of state-anxiety at 26 weeks gestation, *n* = 12; past history of miscarriage, *n* = 53; and, mode of infant feeding at 1st month, *n* = 2. These values were assumed to be missing at random and missing values were predicted by multiple imputation. For this study, 100 imputations were created and results were pooled. To ensure that results were not affected by imputation, the same statistical analyses were done only for participants with complete data (*n* = 430) and compared with results of imputed data. In sensitivity analyses, the models were further adjusted for history of psychological suffering, marital status, pregnancy BMI, alcohol consumption, smoking and smoke exposure before and during pregnancy, exercise extent and employment status during pregnancy, mode of delivery, gestational age, and availability of social support during confinement. All of the statistical analyses were conducted using Statistical Package for the Social Sciences software package version 23 (IBM Corp., Armonk, NY, USA) and *p* < 0.05 was considered statistically significant. 

## 3. Results

### 3.1. Postnatal Dietary Patterns

Four dietary patterns followed during the confinement period were identified (Table 1). The Traditional-Chinese-Confinement (TCC) diet was characterized by high consumption of traditional dried fruits, herbal tea, rhizomes, Chinese herbs, and foods cooked with wine, alcohol, or vinegar, which are considered “hot” foods and traditionally consumed by mothers who observe the traditional Chinese confinement diet during the 1st month postpartum [26,28,42,43]. The Traditional-Indian-Confinement (TIC) diet was characterized by ethnic bread, Indian herbs, whole milk, seed herbs, and butter/ghee, which are known to be common food items present in diets across Northern and Southern India. Whole milk, butter, ghee, garlic, and certain herbs, such as ajwain and fenugreek, are considered key foods during the traditional Indian confinement period and are typically consumed in greater quantities during this period [27,31,44,45,46]. The Eat-Out diet comprised of deep-fried/mashed potato, sweetened and cordial drinks, ice-cream, chips/crisps, deep-fried dim sum, and local savoury snacks, which reflected typical food items sold at fast food restaurants or purchased outside the home. Lastly, the Soup-Vegetables-Fruits (SVF) diet was characterized by higher intakes of assorted soup (vegetables, meat, fish, seafood, and noodles), vegetables, fish (non-fried), and fresh fruits, and lower intakes of sweet spreads and milk-based drinks. This dietary pattern resembles the “healthy” or “health-conscious” dietary pattern reported in other literature with common elements, such as high consumption of vegetables, fruits, fish (non-fried), whole grains, low-fat milk, and dairy products and low consumption of snacks and meat products [47]. 

### 3.2. Characteristics of Participants in Relation to Adherence to Postnatal Dietary Patterns

The prevalence of probable PPD (EPDS score ≥ 13) was 9.8% and mean (SD) EPDS score at three months postpartum 6.05 (4.66). For state-anxiety symptoms, the mean (SD) STAI-state subscale score was 33.83 (10.14). Table 2 summarizes the main characteristics of participants according to tertiles of dietary patterns scores. More characteristics of participants were examined and presented in Supplementary Table S3. Participants who adhered to Traditional-Chinese-Confinement diet were older, predominantly Chinese, had higher education status and were less likely to have developed probable depression during pregnancy. They were also less likely to smoke or be exposed to smoke, but more likely to have consumed alcohol before and during pregnancy. Participants who scored higher for the Soup-Vegetables-Fruits diet shared similar characteristics to those who adhered to Traditional-Chinese-Confinement diet except that they tended to be nulliparous, and were less likely to formula feed their child for the first month. They were more likely to exercise for ≤150 min/week, to be employed, and tended to have less symptoms of state-anxiety at 26 weeks gestation. Greater adherence to Traditional-Indian-Confinement diet was observed in older, multiparous participants who were likely to be of Indian ethnicity, had higher education, and exclusively breastfeed their newborn for the first month postpartum. These participants tended to be unemployed at 26 weeks gestation and were less likely to have consumed alcohol, smoked, or exposed to smoke before and during pregnancy. Participants who scored higher for Eat-Out diet were less likely to be of Chinese ethnicity and tended to exclusively breastfeed their infant (*p* < 0.05 for all). 

### 3.3. Associations between Postnatal Dietary Patterns and Postpartum Mental Health

In multivariable models, greater adherence to Traditional-Indian-Confinement diet was associated with less PPD symptoms (β = −0.62 EPDS scores per SD increase in TIC score; 95% CI = −1.16, −0.09) and trend association with a lower likelihood of probable PPD (OR = 0.56; 95% CI = 0.31, 1.01) (Table 3). A non-significant trend between the Soup-Vegetables-Fruits diet score and less PPD symptoms was also observed (β = −0.48 EPDS scores per SD increase in SVF score; 95% CI = −0.97, 0.02). A higher Soup-Vegetables-Fruits dietary pattern score was significantly associated with less PPA symptoms (β = −1.49 STAI-state subscale scores per SD increase in SVF score; 95% CI = −2.56, −0.42). No associations were observed for Traditional-Chinese-Confinement and Eat-Out diets in relation to postpartum mental health.

### 3.4. Sensitivity Analyses

When the models were further adjusted for other maternal covariates, the main results of the analyses remained largely similar. The Traditional-Indian-Confinement dietary pattern score was inversely associated with both continuous EPDS scores (β = −0.67; 95% CI = −1.21, −0.14) and probable PPD (OR = 0.52; 95% CI = 0.28, 0.96). The Soup-Vegetables-Fruits diet score remained inversely associated with symptoms of state-anxiety at three months’ postpartum (β = −1.30, 95% CI = −2.38, −0.22). When the analyses were restricted to participants with no missing data (*n* = 430), the associations between Traditional-Indian-Confinement diet and PPD symptoms (β = −0.57, 95% CI = −1.11, −0.01) and probable PPD (OR = 0.63; 95% CI = 0.34, 1.16), and association between Soup-Vegetables-Fruits diet and PPA symptoms (β = −1.47, 95% CI = −2.60, −0.34) were generally similar.

## 4. Discussion

In this study, we observed the emergence of four dietary patterns when examining postnatal diets in a multi-ethnic Asian cohort. When relating them to maternal mental health status at three months postpartum, the Traditional-Indian-Confinement diet was found to be associated with less PPD symptoms. The Soup-Vegetables-Fruits diet also showed a non-significant trend towards an inverse association with PPD symptoms and was associated with less PPA symptoms. 

### 4.1. Association between Traditional-Indian-Confinement Diet and PPD

The Traditional-Indian-Confinement diet, characterized by common food groups present in traditional confinement diets adhered by mothers across India [27,44] is shown to be inversely associated with PPD symptoms. To our knowledge, no dietary pattern similar to Traditional-Indian-Confinement pattern has been examined in relation to PPD in previous literature for direct comparison. However, key food groups of Traditional-Indian-Confinement diet, such as Indian herbs and seed herbs (e.g., saffron [48,49], fenugreek [50]), as well as curcumin [51,52] present in curry dishes have previously been reported to be effective in alleviating depressive symptoms via their anti-inflammatory [49,53], neuroprotective [54], serotonergic [55,56], and hypothalamic pituitary adrenal (HPA) regulation effects [57]. The Traditional-Indian-Confinement diet also comprises of legumes and pulses and ethnic bread (e.g., chapatti, thosai, idli, and naan), which are rich in B-group vitamins, are crucial for the synthesis of monoamine neurotransmitters and may have helped to establish protection against PPD symptoms [20,58,59]. Taken together, these combined pieces of evidences help explain and substantiate our findings that Traditional-Indian-Confinement diet might be protective of PPD symptoms.

### 4.2. Association between Soup-Vegetables-Fruits Diet and Maternal Postpartum Mental Health

Adherence to Soup-Vegetables-Fruits diet, which is conceptually similar to a healthy dietary pattern [47], was associated with less PPA symptoms and a trend towards less PPD symptoms. Our results are in line with the Rhea cohort study that reported an association between adherence to “health conscious” dietary pattern during pregnancy and less PPD symptoms [19]. Increased intake of fruits and vegetables was also observed to be associated with less psychological distress and lower risk of depression in a longitudinal study of over 6000 Canadians above 18 years of age [60]. In relation to PPA, our results are in accordance with two previous studies that reported that adherence to dietary patterns characterized by high intake of vegetables, fruits, fish, meat and dairy products during pre-pregnancy or pregnancy are associated with lower risk of anxiety during pregnancy and postpartum, respectively [17,22]. While few studies have examined the association between maternal dietary patterns and anxiety symptoms, our findings provided further insights that adherence to a healthy dietary pattern, such as a Soup-Vegetables-Fruits dietary pattern during postpartum might be protective of symptoms of PPA. 

A possible mechanism through which a Soup-Vegetables-Fruits dietary pattern might protect against symptoms of depression and anxiety is through regulating oxidative damage that is caused by oxidative stress [61,62]. The pattern is characterized by high intake of vegetables and fruits, which are rich in antioxidants, such as Vitamin C, Vitamin E, and β-carotene [63]. As antioxidants help to counterbalance the effect of free radical oxidants, reduce oxidation damage, and provide neural protection [64], this could therefore explain the protective effect of a Soup-Vegetables-Fruits dietary pattern against mental health disorders. Furthermore, Soup-Vegetables-Fruits diet is high in fish (non-fried), some of which is rich in n-3 fatty acids. N-3 fatty acids play a significant role in maintaining the fluidity and permeability of neuronal membranes, regulating the function and metabolism of serotonin, and also have properties of anti-inflammation and anti-oxidation [59]. Maternal n-3 fatty acids levels, particularly DHA, are depleted during pregnancy and a low n-3 fatty acids level has been associated with PPD [11]. Adherence to a Soup-Vegetables-Fruits dietary pattern could therefore supplement mothers with adequate amount of nutrients and provide protection against PPD and PPA. 

### 4.3. Strengths

To our knowledge, this is the first prospective study to derive unique Asian dietary patterns followed during confinement period in a multi-ethnic cohort and to draw associations between data-driven postnatal dietary patterns and maternal mental health outcomes. The use of data-driven approach in deriving dietary patterns has allowed us to discover particular postnatal dietary patterns in Asia. Additionally, whilst we observed that multiparous mothers were less likely to be depressed or anxious when compared to nulliparous mothers, we adjusted for this potential confounding factor (parity) and other key confounders factors, such as ethnicity, maternal age, education level, history of miscarriage, maternal mental health status (EPDS and STAI-state scores) at 26 weeks gestation, and mode of infant feeding during 1st month postpartum. In all, our study contributes to existing knowledge of diet and postpartum mental health status by providing evidence that diet at postnatal time-point is also associated with maternal mental health three months postpartum, in addition to antenatal diets described in previous literature. 

### 4.4. Limitations

A number of study limitations need to be considered. Firstly, the findings might not be representative of Singapore population due to the relatively small sample size of this study. A larger sample size may be required to extrapolate these results to the population of Singapore. Secondly, as data-driven approach was used, dietary patterns that were derived in this study were specific to Asia’s context. As a result, findings in relation to these Asian-specific dietary patterns (such as Traditional-Indian-Confinement diet and Traditional-Chinese-Confinement diet) cannot be simply extrapolated to other populations. These Asian-specific dietary patterns may also render comparison with results from other studies difficult. However, major food groups of Eat-Out diet and Soup-Vegetables-Fruits diet shared similarity with widely replicated and recognized dietary patterns, such as western diet and prudent/healthy diet, respectively [65]. Furthermore, our results are in line with previous studies that investigated the association between major food groups of other dietary patterns and mental health outcomes. 

We recognized that it is possible that mothers who adhered to their ethnic dietary patterns may be more likely to have greater cultural identity, community, and social support, which in itself could provide protective effects against PPD. For instance, these mothers may also receive stronger family support with regards to infant care, which can be a key source of postnatal stress. We, however, did not have information on number of family members (reflecting family support) or type of postpartum support to confirm this. In contrast, we did not observe any associations between diet and mental health with mothers following the traditional Chinese confinement diet, suggesting that following traditional cultural practices may not necessarily translate to stronger family and social support. Confinement practices often include behavioral restrictions that could affect participants’ diet and risk of developing PPD. For instance, most mothers following confinement are required to stay at home throughout the confinement period. This could have resulted in them consuming mostly home-cooked meals. Some are less able to cope with these confinement restrictions and may end up more vulnerable to the risk of developing PPD [66]. This may also explain why mothers who chose not to follow traditional confinement diets, such as those adhering to the Soup-Vegetables-Fruits diet, tend to be associated with lesser anxiety at postpartum. Taken together, we acknowledge that traditional/cultural practices, which we were not able to fully account for, could have confounded our findings. 

## 5. Conclusions

Our study derived particular postnatal dietary patterns present in a multi-ethnic Asian cohort and established associations with maternal mental health status at three months postpartum. The Traditional-Indian-Confinement diet was inversely associated with symptoms of PPD (and trend association with reduced probable PPD (EPDS ≥ 13)), while Soup-Vegetables-Fruits diet was shown to be protective of PPA symptoms. We provided further evidence that postnatal diet could have an effect on postpartum mental health and hypothesize that a balanced diet with emphasis on fruits, vegetables, fish, and legumes during first month postpartum could be a potential preventive measure against postpartum mental health disorders. Further studies are warranted to confirm these findings. 

## Figures and Tables

**Table 1:** Factor loadings for maternal dietary patterns during the confinement period (*n* = 490) ^1^.

Food Groups	TCC Diet	TIC Diet	Eat-Out Diet	SVF Diet
**Traditional dried fruits ^2,3^**	0.70			
**Herbal tea**	0.67			
**Rhizomes ^2^**	0.64			
**Traditional Chinese herbs ^2,4^**	0.56			
**Foods cooked with wine or alcohol ^2^**	0.56			
**Herbal-based soup**	0.50			
**Pig trotter/tail/skin dishes**	0.50			
**Sesame oil ^2^**	0.46			
**Poultry (NF)**	0.45			
**Foods cooked with vinegar ^2^**	0.43			
**Red meat (NF)**	0.34			
**Ethnic bread ^5^**		0.64		
**Indian herbs ^2,6^**		0.58		
**Whole milk**		0.49		
**Seed herbs ^2,7^**		0.48		
**Butter/ghee**		0.47		
**Curry-based gravies**		0.46		
**Legumes/pulses**		0.44		
**Allium ^2^**		0.43		
**Coffee and tea**		0.35		
**Garlic ^2^**		0.35		
**White bread**		0.30		
**Deep-fried/mashed potato**			0.48	
**Sweetened and cordial drinks ^8^**			0.46	
**Ice-cream**			0.41	
**Chips/crisps**			0.40	
**Dim sum and local savoury snacks (F)**			0.38	
**Meat (F)**			0.38	
**Soya bean drinks**			0.36	
**Flavoured rice**			0.33	
**Burgers**			0.32	
**Pizza**			0.31	
**Flavoured noodles**			0.31	
**Carbonated drinks**			0.30	
**Vegetable/meat/fish/seafood soup**				0.53
**Noodles (in soup)**				0.38
**Vegetables—Cruciferous, Leafy, Yellow/Orange/Red**				0.37
**Other vegetables ^9^ and stir fried/boiled potato**				0.35
**Fish (NF)**				0.35
**Sweet spreads**				−0.33
**Fresh fruits**				0.33
**Milk-based drinks**				−0.30

F = Deep-fried or cooked in curry/coconut based gravies; NF = Stir-fried, pan-fried, braised, stewed, boiled, steamed, grilled, baked or roasted preparation; ^1^ Values are factor loadings derived from exploratory factor analysis of 84 food groups. Only absolute values ≥ 0.3 were presented. TCC: Traditional Chinese Confinement; TIC: Traditional Indian Confinement; SVF: Soup, Vegetables and Fruits; ^2^ Frequency of food intake was recorded; ^3^ Dates, longan, and wolfberry; ^4^ Dang gui, dang shen, pakkei, and ginseng; ^5^ Steamed buns, chapatti, thosai, idli, and naan; ^6^ Ayurveda, confinement herbs, and spices; ^7^ Cumin seed, fenugreek, and coriander; ^8^ Non-carbonated sweetened beverages, cordial, and sweetened fruit juice; ^9^ Starchy, fruit-bearing vegetables, and beans.

**Table 2:** Characteristics of participants by tertiles of dietary pattern scores (*n* = 490) ^1^.

Characteristics	Traditional Chinese Confinement Diet				Traditional Indian Confinement Diet				Eat-Out Diet				Soup, Vegetables and Fruits Diet			
	T1	T2	T3	*p*-Trend	T1	T2	T3	*p*-Trend	T1	T2	T3	*p*-Trend	T1	T2	T3	*p*-Trend
Mother’s age, years	30.3 ± 5.2	31.4 ± 4.8	32.3 ± 4.3	**<0.001**	30.9 ± 5.1	30.9 ± 4.7	32.2 ± 4.6	**0.01**	31.2 ± 4.7	31.8 ± 4.6	31.0 ± 5.2	0.49	30.2 ± 5.2	31.4 ± 4.9	32.4 ± 4.0	**<0.001**
Ethnicity, %				**<0.001**				**<0.001**				**<0.001**				**<0.001**
Chinese	15	36	49		40	35	26		38	37	26		21	33	46	
Malay	72	21	7		33	43	25		26	25	50		58	33	10	
Indian	52	43	5		3	12	85		26	32	42		51	35	14	
Education, %				**<0.001**				**0.01**				0.53				**<0.001**
Primary and Secondary	46	30	23		40	37	23		40	22	38		60	32	8	
Post-sec	34	35	31		31	37	32		31	35	34		42	35	23	
University	26	34	40		31	29	39		32	38	30		12	33	55	
Nulliparous,%	32	37	31	0.6	36	37	28	**0.02**	34	34	32	0.62	26	35	40	**0.001**
Probable depression at 26 weeks gestation, %	56	22	22	**0.03**	39	31	30	0.53	37	40	22	0.22	45	37	18	0.07
High level of state anxiety at 26 weeks gestation, %	42	29	28	0.06	35	41	24	0.05	31	35	34	0.67	49	31	20	**<0.001**
Past history of miscarriage, %	31	38	32	0.68	35	41	24	0.15	35	33	32	0.37	23	37	40	0.08
Mode of infant feeding at 1st Month, %				0.06				**<0.001**				**0.02**				**<0.001**
Exclusively Breastfeed	32	31	36		24	31	46		25	35	40		24	33	43	
Partially Breastfeed	32	33	34		36	35	29		34	36	30		32	34	33	
Formula Feed	44	40	16		47	31	21		51	14	34		70	27	3	

^1^ Values presented are mean ± SDs unless otherwise stated. *p*-trends were assessed by modelling median values of each tertile in linear regression analysis for continuous variables and through Cochran-Mantel-Haenszel chi-square test for linear trends for categorical variables. Number of missing values: *n* = 5 (1.02%) for “Education”, *n* = 9 (1.84%) for “probable depression at 26 weeks gestation”, *n* = 12 (2.45%) for “high level of state anxiety at 26 weeks gestation”, *n* = 53 (10.82%) for “past history of miscarriage”, *n* = 2 (0.41%) for “Mode of infant feeding at 1st Month”.

**Table 3:** Associations between maternal postnatal dietary patterns and mental health outcomes at three months postpartum (*n* = 490) 1.

Dietary Patterns	EPDS Scores		Probable PPD (EPDS ≥ 13) (*n* = 48) 2		STAI-State Subscale Scores	
	*β* (95% CI)	*p*	OR (95% CI)	*p*	*β* (95% CI)	*p*
Traditional Chinese Confinement diet	0.38 (−0.11 to 0.87)	0.13	1.3 (0.85 to 2.00)	0.22	−0.34 (−1.40 to 0.73)	0.54
Traditional Indian Confinement diet	−0.62 (−1.16 to −0.09)	**0.02**	0.56 (0.31 to 1.01)	0.05	−0.51 (−1.66 to 0.64)	0.39
Eat Out diet	−0.04 (−0.44 to 0.36)	0.84	0.87 (0.59 to 1.29)	0.49	0.06 (−0.80 to 0.93)	0.89
Soup, Vegetables and Fruits diet	−0.48 (−0.97 to 0.02)	0.06	0.92 (0.58 to 1.45)	0.73	−1.49 (−2.56 to −0.42)	**0.006**

^1^ Values are linear regression coefficients (95% CI) for EPDS and STAI-state scores or logistic regression coefficients (95% CI) for probable PPD per 1 SD increment of maternal dietary pattern scores. Models are adjusted for maternal age, ethnicity, parity, education level, history of miscarriage, probable depression of mothers at 26 weeks gestation (for EPDS scores and probable PPD models), STAI-state score at 26 weeks gestation (for STAI-state subscale scores model) and mode of infant feeding during 1st month postpartum. EPDS: Edinburgh Postnatal Depression Scale; STAI: State-Trait Anxiety Inventory. ^2^ Probable PPD defined as EPDS scores measured at three months postpartum ≥ 13.

## Supplementary Materials

The following are available online at http://www.mdpi.com/2072-6643/10/3/299/s1, Table S1, List of 84 food groups. Table S2, Characteristics of eligible participants included in study and those who did not participate. Table S3, Additional characteristics of participants by tertiles of dietary pattern scores (*n* = 490).
