# Supplementary material for: Prospective Associations of Maternal Dietary Patterns and Postpartum Mental Health in a Multi-Ethnic Asian Cohort: The Growing up in Singapore towards Healthy Outcomes (GUSTO) Study
Source: Nutrients. 2018 Mar 2;10(3):299. doi: 10.3390/nu10030299 (PMC5872717; doi:10.3390/nu10030299)
Supplement: Supplementary file 1 [file nutrients-10-00299-s001.pdf]

nutrients

MDPI

Supplementary Materials: Prospective Associations of Maternal Dietary Patterns and Postpartum Mental Health in a Multi-ethnic Asian Cohort: the Growing Up in Singapore Towards Healthy Outcomes (GUSTO) Study

Cherlyen Teo, Ai-Ru Chia, Marjorelee T. Colega, Ling-Wei Chen, Doris Fok, Wei Wei Pang, Keith M. Godfrey, Kok Hian Tan, Fabian Yap, Lynette Pei-Chi Shek, Yap-Seng Chong, Michael Meaney, Helen Chen, Mary Foong-Fong Chong *

Table S1. List of 84 food groups.

Food Groups	
Poultry (NF)	Soya bean drinks
Red meat (NF)	Coffee and tea
Meat (F)	Herbal tea
Pig trotter/tail/skin dishes	Alcoholic drinks
Meat products and dried and preserved meat	Essence drinks
Other meat and innards	Dessert soup
Fish (NF)	Kueh, pastries, biscuits, cookies
Fish (F)	Dim sum and local savoury snacks (NF)
Seafood	Dim sum and local savoury snacks(F)
Dried and preserved seafood and fish products	Ice-cream
Eggs	Chips/crisps
Vegetables-Cruciferous, Leafy, Yellow/Orange/Red	Blended oil
Other vegetables and stir fried/boiled potato	PUFA/MUFA oil
Deep-fried/mashed potato	Butter/ghee
Fresh fruits	Margarine and peanut butter
Fresh fruit juice	Other oils and fats (includes salad dressing and mayonnaise)
Fruits, canned/dried and preserved	Cream/condensed/powder type soup
Bean curd	Vegetable/meat/fish/seafood soup
Legumes/pulses	Herbal-based soup
Nuts and oily seeds	Tomato-based gravies
White rice	Cream-based gravies
Brown/red rice	Curry-based gravies
Flavoured rice	Soya sauce based gravies
Porridge	Others sauces/gravies
Other grains	Sugar, honey, syrup, condensed milk
Breakfast cereals and cereal bars	Sweet spreads
Noodles (in soup)	Sweets and chocolates
Flavoured noodles	Burgers
Pasta	Pizza
White bread	Other fast food items
Wholemeal/multigrain bread	Food cooked with wine or alcohol*

Ethnic bread	Food cooked with vinegar*
Bread with sweet/savoury fillings	Sesame oil*
Low-fat/ skimmed milk	Traditional dried fruits*
Whole milk	Traditional Chinese herbs*
Formula milk	Malay/Indonesian herbs
Milk-based drinks	Indian herbs*
Yoghurt and cultured drinks	Allium*
Full cream/ low-fat cheese	Garlic*
Milk (others)	Rhizomes*
Carbonated drinks	Seed herbs*
Sweetened and cordial drinks	Dried herbs*

* Frequency of food intake was recorded. F = Deep-fried or cooked in curry/coconut based gravies; NF = Stir-fried, pan-fried, braised, stewed, boiled, steamed, grilled, baked or roasted preparation.

Table S2. Characteristics of eligible participants included in study and those who did not participate.[1]

Characteristics of study participants	Included (*n* = 490)	Not included (*n* = 759)	*P*
Maternal Age, years	31.4 ± 4.8	30.1 ± 5.3	<0.001
Parity, *n* (%)			
0	222 (45.3)	320 (46.1)	0.830
>0	268 (54.7)	374 (53.9)	
Ethnicity, *n* (%)			<0.001
Chinese	312 (63.7)	386 (50.9)	
Malay	113 (23.1)	212 (27.9)	
Indian	65 (13.3)	161 (21.2)	
Education, *n* (%)			<0.001
Primary and Secondary	110 (22.7)	259 (34.9)	
Post-secondary	166 (34.2)	262 (35.3)	
University	209 (43.1)	222 (29.9)	
Pregnancy BMI, kg/m^2	25.9 ± 4.2	26.4 ± 4.63	0.045
Mode of infant feeding at 1st Month, *n*(%)			<0.001
Exclusively Breastfed	140 (28.7)	126 (21.3)	
Partially Breastfed	304 (62.3)	373 (63.1)	
Formula Fed	44 (9)	92 (15.6)	

[1]Values presented are mean ± SD or *n* (%). *P*-values are determined by independent T-test (continuous variables) and chi-square analysis (categorical variables). Number of missing data for Parity (*n* = 64), Education (*n* = 19), Pregnancy BMI (*n* = 87), mode of infant feeding during 1st month (*n* = 168).

Table S3. Additional characteristics of participants by tertiles of dietary pattern scores (n = 490).[1]

	**Traditional Chinese Confinement Diet**				**Traditional Indian Confinement Diet**				**Eat-Out Diet**				**Soup, Vegetables and Fruits Diet**			
	T1	T2	T3	*p*-Trend	T1	T2	T3	*p*-Trend	T1	T2	T3	*p*-Trend	T1	T2	T3	*p*-Trend
Maternal Characteristics																
Pregnancy BMI, kg/m^2	27.0 ± 4.7	26.0 ± 4.1	24.7 ± 3.2	<0.001	25.7 ± 4.2	25.8 ± 4.2	26.2 ± 4.0	0.31	25.7 ± 4.3	25.2 ± 4.8	26.8 ± 4.2	0.004	26.6 ± 4.5	26.2 ± 4.4	24.8 ± 3.2	<0.001
Married, %	33	33	34	0.02[2]	33	34	34	0.18[2]	33	34	33	0.47[2]	33	33	34	0.35[2]
Planned pregnancy, %	31	30	39	0.02	30	33	37	0.07	32	36	32	0.65	27	31	43	<0.001
Lifestyle Factors																
Smoked or exposed to smoke before or during pregnancy, %	44	32	24	<0.001	38	34	27	0.01	38	28	35	0.74	49	34	16	<0.001
Alcohol consumption before or during pregnancy, %	25	29	46	<0.001	40	32	28	0.03	33	35	32	0.73	22	38	40	<0.001
Exercise extent during pregnancy, %				0.35				0.19				0.05				0.03
No exercise	39	38	23		32	39	30		52	32	16		49	23	29	
Only gentle exercise	35	31	33		35	33	32		35	30	34		36	34	30	
≤150mins moderate exercise/week	24	39	37		35	31	34		21	45	34		18	38	44	
>150mins moderate exercise/week	40	30	30		19	39	42		34	27	38		42	25	33	
Employed at 26 weeks gestation, %	32	33	35	0.33	35	35	30	0.02	32	34	34	0.25	30	34	36	0.005
Medical History																
GDM, %	28	27	45	0.01	28	34	38	0.17	24	35	41	0.03	35	29	36	0.7
Past history of stillbirth, %	27	48	25	0.48	37	45	19	0.11	39	47	14	0.03	14	40	47	0.02
Past history of abortion, %	31	50	19	0.12	40	40	20	0.05	41	35	24	0.1	31	37	31	0.33
History of psychological suffering, %	20	52	28	0.66[2]	42	20	38	0.62[2]	62	10	30	0.41[2]	12	52	38	0.42[2]
Characteristics at delivery and postpartum																
Preterm birth,%	35	35	30	0.78	52	26	22	0.09	17	43	39	0.27	39	43	17	0.17
Caesarean delivery, %	33	39	28	0.33	36	32	33	0.68	33	34	33	0.93	35	37	28	0.24
Social support for confinement (Yes), %	31	34	34	0.002	34	33	33	0.74	34	34	32	0.05	32	33	35	0.002
Practice of confinement, %	33	33	34	0.23	33	34	32	0.29	34	34	32	0.08	32	34	34	0.08

[1] Values presented are mean ± SDs unless otherwise stated. *P*-trends were assessed by modelling median values of each tertile in linear regression analysis for continuous variables and through Cochran-Mantel-Haenszel chi-square test for linear trends for categorical variables. Number of missing values: n = 10 (2.04%) for "Pregnancy BMI", n = 9(1.84%) for "married", n = 9 (1.84%) for "smoked or exposed to smoke before or during pregnancy", n = 8 (1.63%) for "Alcohol consumption before or during

pregnancy", $n = 3$ (0.61%) for "exercise extent during pregnancy", $n =$ (1.02%) for "Employed at 26 weeks gestation", $n = 37$ (7.55%) for "planned pregnancy", $n = 58$ (11.84%) for "GDM", $n = 53$ (10.82%) for "past history of stillbirth", $n = 53$ (10.82%) for "past history of abortion", $n = 6$ (1.22%) for "history of psychological suffering", $n = 2$ (0.41%) for "practice of confinement", $n = 35$ (7.14%) for "support for confinement". [2] Tests may not be valid due to violation of chi-square assumption, i.e. more than 20% of the cells have expected cell counts less than 5
